# Mitochondrial Glutathione in Cellular Redox Homeostasis and Disease Manifestation

**DOI:** 10.3390/ijms25021314

**Published:** 2024-01-21

**Authors:** Tsung-Hsien Chen, Hsiang-Chen Wang, Chia-Jung Chang, Shih-Yu Lee

**Affiliations:** 1 Department of Internal Medicine, Ditmanson Medical Foundation Chia-Yi Christian Hospital, Chiayi 60002, Taiwan; cych13794@gmail.com; 2 Department of Mechanical Engineering, National Chung Cheng University, Chiayi 62102, Taiwan; hcwang@ccu.edu.tw; 3 Division of Critical Care Medicine, Department of Internal Medicine, Ditmanson Medical Foundation Chia-Yi Christian Hospital, Chiayi 60002, Taiwan

**Keywords:** glutathione, mitochondria, oxidative phosphorylation, reactive oxygen species (ROS), programmed cell death, GSH deficiency, GSH/GSSG

## Abstract

Mitochondria are critical for providing energy to maintain cell viability. Oxidative phosphorylation involves the transfer of electrons from energy substrates to oxygen to produce adenosine triphosphate. Mitochondria also regulate cell proliferation, metastasis, and deterioration. The flow of electrons in the mitochondrial respiratory chain generates reactive oxygen species (ROS), which are harmful to cells at high levels. Oxidative stress caused by ROS accumulation has been associated with an increased risk of cancer, and cardiovascular and liver diseases. Glutathione (GSH) is an abundant cellular antioxidant that is primarily synthesized in the cytoplasm and delivered to the mitochondria. Mitochondrial glutathione (mGSH) metabolizes hydrogen peroxide within the mitochondria. A long-term imbalance in the ratio of mitochondrial ROS to mGSH can cause cell dysfunction, apoptosis, necroptosis, and ferroptosis, which may lead to disease. This study aimed to review the physiological functions, anabolism, variations in organ tissue accumulation, and delivery of GSH to the mitochondria and the relationships between mGSH levels, the GSH/GSH disulfide (GSSG) ratio, programmed cell death, and ferroptosis. We also discuss diseases caused by mGSH deficiency and related therapeutics.

## 1. Introduction 

Mitochondria play a vital role in oxidative phosphorylation (OXPHOS), which generates adenosine triphosphate (ATP) for energy storage and release. The electron transport chain (ETC) facilitates electron and proton transfer, creating a proton gradient that drives ATP synthesis. Mitochondria are dynamic organelles that consume oxygen and produce reactive oxygen species (ROS) via the mitochondrial respiratory chain [1]. Mitochondrial defects lead to reduced ATP production, impaired mitochondrial respiration, and increased production [2]. Mitochondrial dysfunction can lead to ATP depletion and excess ROS, which, in turn, activate harmful cellular pathways [3]. Mutation-induced mitochondrial dysfunction can lead to diseases that directly affect cellular metabolism. In addition, mitochondria are an energy source for cancer cells and are involved in the regulation of cell proliferation, metastasis, and deterioration. Furthermore, mitochondria can adapt to tumor conditions and may evolve into “oncogenic mitochondria” that transfer malignant capabilities to recipient cells [4]. Mitochondrial ROS (mtROS) can be produced during the Krebs cycle or ETC [5], increasing ROS levels in cancer cells due to increased metabolic activity and altered antioxidant capacity [6], stimulating cancer onset and progression [7]. Within the respiratory chain, the main site for generating superoxide anions is the ubiquinone pool of OXPHOS complex III, where a single electron transfer to molecular oxygen occurs. Superoxide anions are then converted to superoxide and hydrogen peroxide (H_2_O_2_) through the action of superoxide dismutase (SOD). These species serve as precursors for generating hydroxyl radicals, with the participation of transition metals [8]. In addition, when mitochondrial DNA and cytochrome c are released from damaged mitochondria into the cytoplasm, they activate damage-associated molecular patterns, which trigger a chronic inflammatory response [9].

Mitochondrial glutathione (mGSH) plays a crucial role in mitochondria. It transports cellular glutathione (GSH) pools from the cytoplasm to the mitochondrial matrix using carriers [8]. It acts as an antioxidant, detoxifier of foreign substances, and stabilizer of mitochondrial DNA [10]. Depletion of mGSH significantly reduces basal mitochondrial respiration and ATP production, highlighting the dependence of mitochondrial function and respiration on mGSH levels [11]. mGSH also serves as a redox regulator for OXPHOS proteins and plays a role in the sequential transfer of electrons between OXPHOS complexes located in the inner mitochondrial membrane [12]. Additionally, mGSH also plays a critical role in supporting redox signaling and the biosynthesis of iron–sulfur (Fe-S) cluster cofactors within the mitochondrial matrix. The regulation of OXPHOS proteins suggests a possible link between mitochondrial metabolism and redox homeostasis, potentially via mGSH status [13].

## 2. Reactive Oxygen Species and Antioxidant Systems

**Figure 1:**
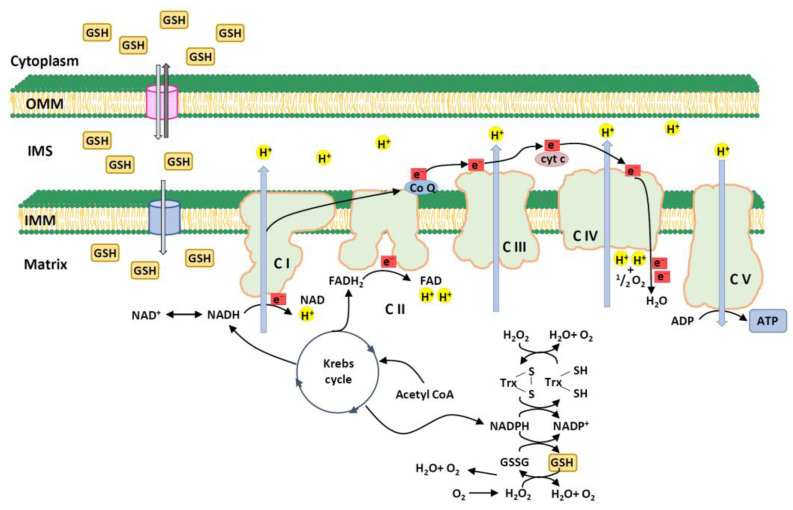
Production of reactive oxygen species via mitochondrial oxidative phosphorylation (OXPHOS) process. OXPHOS consists of five protein complexes (I, II, III, IV, and V) and two electron carriers (CoQ and cyt c). Complex I accepts electrons from NADH and transfers them to CoQ. Complex II receives electrons from succinate and transfers them to CoQ. Complex III transfers electrons from CoQ to cyt c. Complex IV transfers electrons from cyt c to molecular oxygen, thereby reducing it to water. Complexes I, III, and IV pump protons through the mitochondrial inner membrane. These electron transfers create a proton gradient that drives the protons back into the mitochondrial matrix via ATP synthase. The leakage of electrons from complexes I and III results in the partial reduction of oxygen to superoxides [14,15]. Other potential ROS production sites include mitochondrial complex II, various mitochondrial enzyme components, and respiratory chain components [16,17]. Acetyl CoA, acetyl coenzyme A; ADP, adenosine diphosphate; ATP, adenosine triphosphate; CI, complex I; CII, complex II; CIII, complex III; CIV, complex IV; CV, complex V; CoQ, coenzyme Q; cyt c, cytochrome complex; FAD, flavin adenine dinucleotide; FADH_2_, reduced form of flavine adenine dinucleotide; GSH, glutathione; GSSG, glutathione disulfide; IMM, inner mitochondrial membrane; IMS, inner mitochondrial space; NAD^+^, oxidized form of nicotinamide adenine dinucleotide; NADH, reduced form of nicotinamide adenine dinucleotide; NADP^+^, oxidized form of nicotinamide adenine dinucleotide phosphate; NADPH, reduced form of nicotinamide adenine dinucleotide phosphate; OMM, outer mitochondrial membrane; Trx, thioredoxin.

Mitochondria are a major endogenous enzymatic source of ROS in mammalian cells, mainly via OXPHOS, which is located on the inner mitochondrial membrane (Figure 1). 

**Figure 2:**
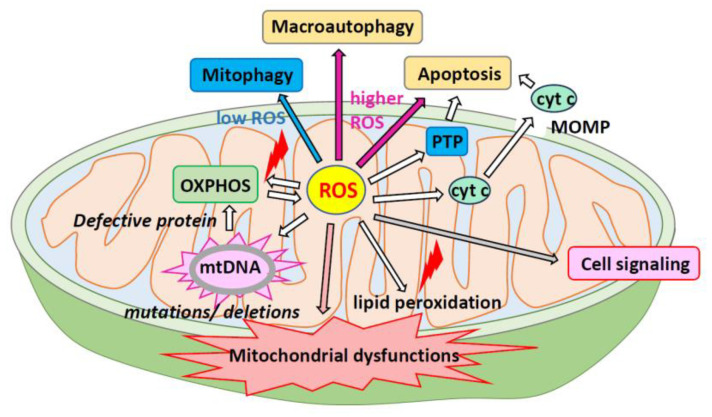
Excessive production of reactive oxygen species results in altered signaling, release of cytochrome c, and cell damage. While ROS play important roles in cell signaling, an imbalance of mtROS can cause lipid peroxidation and disrupt mitochondrial function. Moreover, this imbalance can result in mutations or deletions in mtDNA, affecting mitochondrial protein function and disrupting OXPHOS. Dysfunctional OXPHOS leads to an increased accumulation of ROS. When ROS levels are low, the mitophagy mechanism is activated, but when ROS levels are high, the apoptosis mechanism is triggered. ROS also induces the release of cyt c and PTP, promoting apoptosis. cyt c, cytochrome c; MOMP, mitochondrial outer membrane permeabilization; mtDNA, mitochondrial DNA; OXPHOS, oxidative phosphorylation; PTP, protein tyrosine phosphatases; ROS, reactive oxygen species.

mtROS serve as important signaling molecules, including superoxide anion radical (^•^O_2_^–^), H_2_O_2_, singlet oxygen (^1^O_2_), and hydroxyl radical (^•^OH) in photocatalysis [18,19] and play a role in cellular metabolism under low-level hypoxia [19]. mtROS are byproducts of the mitochondrial ETC [20,21] and can cause oxidative damage to cellular components, including proteins, lipids, and DNA. As mtROS production increases, the components of the respiratory chain and enzymes of the Krebs cycle are inactivated, leading to cell structure damage [22]. Excessive ROS can harm mitochondria and affect the redox status of the entire cell (Figure 2). As local ROS are released from the mitochondria into the cytoplasm, ROS production can have long-term effects on mitochondrial morphology and ROS homeostasis [23]. Excessive mitochondrial fusion and motility can lead to changes in the cell-wide redox status [23]. Elevated levels of mtROS can trigger programmed cell death pathways such as the apoptosis/autophagy pathway [24].

Most mitochondrial antioxidant enzymes are produced in the cytoplasm and transported to the mitochondrial matrix [25]. SOD is an important antioxidant enzyme involved in the first line of intracellular defense against ROS. SOD is rapidly converted into H_2_O_2_ by two dismutases, manganese-dependent superoxide dismutase (MnSOD; SOD2 protein) in the mitochondrial matrix and copper–zinc superoxide dismutase (CuZnSOD; SOD1 protein) in the mitochondrial intermembrane space [26,27]. MnSOD reduces the ^•^O_2_^–^ of ROS to H_2_O_2_. MnSOD is regulated by sirtuin-3 (which mediates its deacetylation). When ETC activity decreases, sirtuin-3-mediated deacetylation of MnSOD decreases, resulting in a decrease in MnSOD activity [28,29]. Peroxynitrite, a powerful oxidant, induces nitration and inactivates MnSOD, thereby exacerbating mitochondrial nitrosative damage [30].

**Figure 3:**
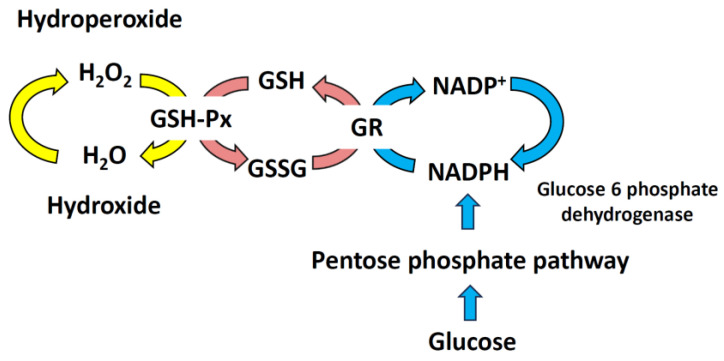
Glutathione is a biological redox buffer. GSH is an important antioxidant in cells as it helps maintain the reduced state of proteins. In a reaction catalyzed by GSH-Px, two GSH molecules form a disulfide bond through sulfhydryl dehydrogenation, resulting in the production of GSSG. Additionally, GR oxidizes NADPH and converts GSSG back into GSH. In normal cells, GSH makes up more than 90% of the total amount of GSH. However, when the level of oxidative stress in cells increases, the content of GSSG rises, causing the ratio of GSH to GSSG to decrease. The ratio of GSH/GSSG reflects the redox capacity of the cell, which is maintained via the oxidation/reduction reactions of GSH-Px and GR. GR, glutathione reductase; GSH, glutathione; GSH-Px, glutathione peroxidase; GSSG, GSH disulfide; NADP^+^, phosphate oxidized form of nicotinamide adenine dinucleotide; NADPH, reduced form of NADP^+^.

Mammalian cells use redox reactions to generate energy and synthesize cellular components from nutrients. The main redox couples involved are nicotinamide adenine dinucleotide in its oxidized and reduced forms (NAD^+^/NADH), nicotinamide adenine dinucleotide phosphate in its oxidized and reduced forms (NADP^+^/NADPH), and GSH/GSH disulfide (GSSG) [31,32]. NAD^+^ acts as an electron acceptor in glycolysis, whereas NADH donates electrons to mitochondrial OXPHOS. NADPH is the main electron source for fatty acid and nucleic acid biosynthesis [31,33]. GSH reductase relies on NADPH, which is oxidized to NADP^+^, forming a redox cycle to prevent oxidative damage [34]. The NADP^+^/NADPH and GSH/GSSG pairs participate in peroxide reduction. NADPH is essential for thioredoxin reductase to convert oxidized thioredoxin-S_2_ (Trx-S_2_) into its active dithiol form Trx-(SH)_2_ [35,36]. The Trx (Cys-Gly-Pro-Cys) system reduces oxidized cysteine groups on proteins by forming a disulfide bond with Trx. Trx utilizes its antioxidant properties via Trx peroxidase, which eliminates ROS by utilizing reducing equivalents. Trx–(SH)_2_ acts as an electron source to regenerate peroxiredoxins, thereby reducing H_2_O_2_ and organic hydroperoxides [36] (Figure 3). Over time, prolonged ROS stimulation can be cleared, but sudden, large amounts of ROS damage can overwhelm the redox mechanism. This can lead to diffusion between compartments and ROS release into the cytoplasm [23].

## 3. Mitochondrial GSH 

In the mitochondria, catalase reduces H_2_O_2_ to H_2_O and O_2_; however, because the catalase content is low, a certain amount of mGSH is needed to maintain the redox balance. The inhibition of the mitochondrial transporter of GSH leads to a decrease in mGSH levels, impairing mitochondrial bioenergetics and ultimately increasing the cell death rate [10,37]. GSSG is the oxidized form of GSH. During the oxidation of GSH to GSSG by GSH-P_X_, H_2_O_2_ is reduced to H_2_O [37,38]. Glutathione reductase then uses NADPH to convert GSSG back into GSH (Figure 3). Under normal physiological conditions, GSH is the main form, accounting for more than 98% of the observed compound, whereas GSSG accounts for less than 1% [34]. Maintaining a balanced GSH/GSSG ratio is critical for overall cellular health and function [39]. The GSH redox system regulates cell growth, development, and oxidative defense [40]. GSH levels decrease with age [41].

### 3.1. GSH Biosynthesis and Metabolism

**Figure 4:**
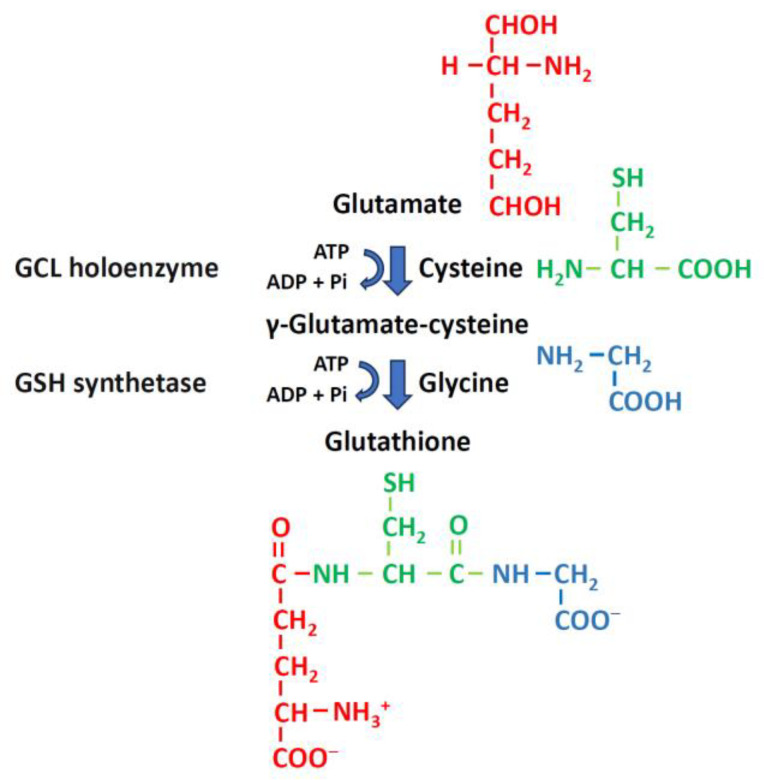
Glutathione synthesis. GSH synthesis relies on the enzymatic activities of cysteine, glutamate, and GSH synthetase (γ-glutamylcysteine ligase). The rate-limiting GSH synthesis intermediate, γ-glutamylcysteine, is formed from glutamate and cysteine and catalyzed by the GCL holoenzyme. The addition of glycine to γ-glutamylcysteine, catalyzed by GSH synthetase, results in the formation of GSH. ATP, adenosine triphosphate; ADP, adenosine diphosphate, GSH, glutathione; GCL, GSH cysteine ligase; Pi, phosphate groups.

**Figure 5:**
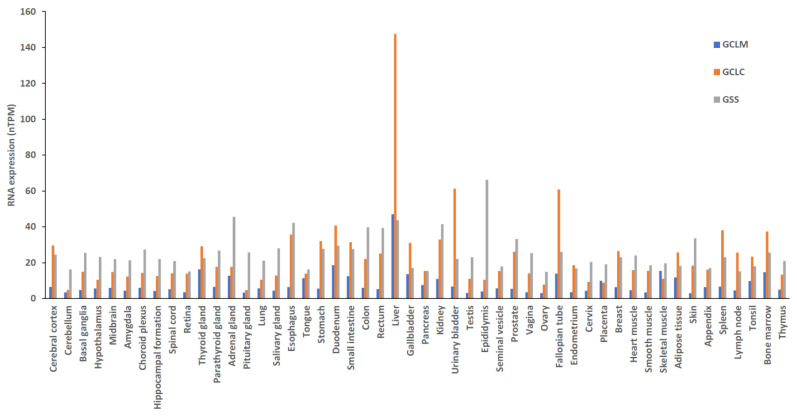
The synthesis of glutathione requires the expression of three genes, namely, GCLC, GCLM, and GSS, in different tissues. These genes (GCLC, GCLM, and GSS) encode the GCL holoenzyme and glutathione synthetase. An overview of the RNA expression reveals that the RNA sequencing (RNA-SEQ) data are a combination of information from the Human Protein Atlas RNA-SEQ data and an internally generated consensus data combination. These datasets were obtained from the Human Protein Atlas Project (https://www.proteinatlas.org (accessed on 6 January 2024). GCLM, glutamate–cysteine ligase regulatory subunit; GCLC, glutamate–cysteine ligase catalytic subunit; GSS, glutathione synthetase; nTPM, transcripts per million.

GSH is a tripeptide composed of glutamic acid, cysteine, and glycine. It is synthesized in the cytoplasm via ATP-dependent steps [42]. GSH synthesis depends on the enzymatic activities of cysteine, glutamate, GSH cysteine ligase (GCL) holoenzyme, and GSH synthetase (γ-glutamylcysteine ligase) (Figure 4) [34]. GSH synthase catalyzes the formation of GSH by adding glycine to glutamylcysteine [34,43]. The GCLC gene and the GCLM gene are responsible for encoding GCL, which catalyzes the formation of glutamylcysteine. GCL is the first rate-limiting enzyme in GSH synthesis. GCL can be produced either by the catalytic subunit GCLC or by the holoenzyme consisting of GCLC and the modifying subunit GCLM. The GSS gene encodes GSH synthase, the second enzyme in the GSH biosynthetic pathway. It catalyzes the condensation of γ-glutamylcysteine and glycine to form GSH. GCLC and GCLM show the highest expression levels in liver tissue, while there is no significant difference in the expression of GSS across various tissues (Figure 5). Thus, the liver is the main source of circulating GSH in the body [34,43]. 

The glutathione-specific γ-glutamyl cyclotransferase family enzymatically degrade GSH [44]. The amino acids produced can be absorbed by the cells and returned to the cells to synthesize GSH. Cysteine–glycine is further broken down into cysteine and glycine by dipeptidases on the cell surface [34,45,46]. Because C-glutamyl transpeptidase is expressed on the outer surface of specific cells [47], the degradation of GSH only occurs in the extracellular space. Intracellular GSH is not easily degraded [34,45]. Furthermore, the accumulation of lactic acid inhibits glucose uptake and glycolysis, leading to cellular ATP depletion and the inhibition of GSH synthesis [48]. In healthy adults, plasma GSH concentrations are 2–5 μM, while GSSG concentrations are 0.14 μM [49,50]. The kidneys are the primary organs responsible for the absorption of plasma GSH. Approximately 80% of the plasma GSH is absorbed by the kidneys, 3/8 of which is rapidly broken down by C-glutamyl transpeptidase and dipeptidase in the renal tubules. GSH efflux from renal tubules is critical for overall GSH transport. After glomerular filtration, absorbed amino acids are used for protein or GSH resynthesis [51].

The cytoplasm contains the highest concentration (approximately 85%) of GSH [52,53]. GSH exists primarily in its reduced form in the cytoplasm. The GSH:GSSG ratio in the cytoplasm is estimated to be approximately 10,000:1–50,000:1 [54]. The concentration of GSH in the cytoplasm can be as high as 10 mM, while the concentration of GSSG is lower. Cytoplasmic GSH is also transported to other organelles. For example, the mammalian peroxisomal membrane allows both GSH and GSSG to pass through. This leads to the intraperoxisomal and cytosolic pools of GSH reaching a redox equilibrium [55]. H_2_O_2_ is controlled via the oxidation of GSH to GSSG and the metabolism of H_2_O_2_ by endoplasmic reticulum (ER)-resident peroxidase-4. The ER requires a highly oxidative environment to carry out its functions [56], so changes in the redox state of the ER significantly affect disulfide bond formation, leading to the oxidation of GSH to GSSG [10]. The ratio of GSH:GSSG in the ER is approximately 1:15 [57]. Although GSH has the lowest concentration in the nucleus, it plays a crucial role in the cell cycle [20,58,59]. Cells preparing to divide have higher nuclear GSH levels [60,61]. During the early stages of cell proliferation, GSH colocalizes with nuclear DNA. GSH regulates DNA replication and maintains the environment of the nuclear structure during the G_1_ phase of mitosis in all eukaryotes [40].

### 3.2. Transport of GSH to Mitochondria

Although mGSH accounts for only 10–15% of cellular GSH [37,62], its concentration (10 mM) is higher than that of cytosolic GSH because of the smaller matrix volume. The cytosol and mitochondrial intermembrane space GSH pools are connected via porins, but there is no significant communication observed between the mitochondrial intermembrane space and matrix pools [63] (Figure 1). The cytosolic GSH reductase system primarily determines the intermembrane space GSH redox potential [63]. GSH can enter the mitochondrial matrix through a voltage-dependent anion channel in the outer mitochondrial membrane. GSH is negatively charged under normal conditions [64]. It cannot pass through the inner mitochondrial membrane because of its negative membrane potential [37,62]. Thus, GSH enters the mitochondrial matrix via active transport or anion exchange. The dicarboxylate carrier and 2-oxoglutarate carrier were identified as the main transporters of GSH [65]. Reduced dicarboxylate carrier performance leads to reduced mGSH levels and impairs mitochondrial complex I activity [66]. The 2-oxoglutarate carrier transfers 2-oxoglutarate as a substitute for dicarboxylic acid [67], which regulates respiration and glycolysis.

**Figure 6:**
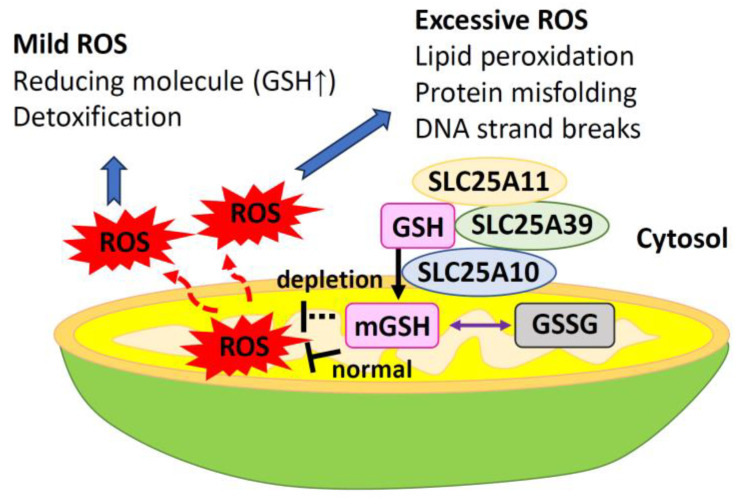
Mitochondrial glutathione, transported from the cytoplasm, can prevent cell damage from reactive oxygen species. GSH is synthesized in the cytosol and can be transported to the mitochondria via specific carriers in the inner mitochondrial membrane. Currently, there are three known carriers for GSH transport: the 2-ketoglutarate carrier (OGC; SLC25A11), the dicarboxylic acid carrier (DIC; SLC25A10), and SLC25A39. When intracellular levels of ROS increase, there is a mild accumulation of ROS that induces the production of reducing molecules, such as GSH, and enhances the detoxification mechanism. However, excessive accumulation of ROS leads to increased lipid peroxidation, protein misfolding, and DNA chain breakage. GSH, glutathione; GSSG, GSH disulfide; mGSH, mitochondrial glutathione; ROS, reactive oxygen species. The ↑ bar indicates an increase.

SLC25A39, a member of the SLC25A family of mitochondrial transporters, is involved in GSH metabolism through its transport activity, and its protein levels are regulated by GSH availability [68,69] (Figure 6). Loss of SLC25A39 leads to a decrease in mGSH import and abundance but does not affect cellular GSH levels [69]. Under normal physiological conditions, SLC25A39 undergoes rapid degradation by the mitochondrial protease AFG3L2 [70]. Depletion of GSH causes AFG3L2 to dissociate from SLC25A39, resulting in a compensatory increase in mGSH uptake [70]. Moreover, SLC25A39 has a dual regulatory mechanism that acts on a mitochondrial transporter protein. This mechanism involves both protein quality control and metabolic sensing [71]. Cells that lack both SLC25A39 and its paralog SLC25A40 show deficiencies in the activity and stability of proteins containing iron–sulfur clusters [69]. SLC25A39 directly controls the level of mGSH in response to iron homeostasis [71]. Additionally, the α-KG carrier (encoded by SLC25A11) and the 2-ketoglutarate carrier (encoded by SLC25A10) also transport cytoplasmic GSH into mitochondria [72] (Figure 6). Salt carriers (encoded by SLC25A1) are potential GSH transporters [38,62]. The glyoxalase system provides an alternative source of mGSH by allowing the intermediate S-d-lactylglutathione to enter the mitochondria and be hydrolyzed by the mitochondrial enzyme glyoxalase II into D-lactate and GSH [73]. 

## 4. The Role of mGSH in Programmed Cell Death

Under conditions of excess ROS, mitochondrial enzyme function becomes impaired and mGSH decreases. Consequently, the mitochondria cannot neutralize the ROS, leading to mitochondrial dysfunction and, ultimately, cell death [74,75] (Figure 6). These processes trigger programmed cell death, leading to apoptosis, autophagy, necroptosis, and ferroptosis [76]. These different forms of cell death may occur simultaneously or sequentially and interact, with one eventually becoming dominant [77]. Consuming mGSH raises ROS levels and uses up ATP, which leads to a shift from apoptosis to necrosis [78]. In addition, GSH indirectly controls the redox state of cardiolipin (an important regulator of apoptosis), thereby determining whether cell death occurs via necrosis or apoptosis [79,80].

### 4.1. Autophagy and Mitophagy in Cellular Damage

ROS-induced autophagy may be a cytoprotective mechanism that alleviates ROS effects or a destructive process. Macroautophagy plays an important role in degrading large aggregates of oxidatively damaged/unfolded proteins that are cleared by the autophagy–lysosomal system. Impaired autophagy leads to protein accumulation [81]. Elevated ROS levels can regulate autophagy via various pathways, such as by activating the adenosine-monophosphate-activated protein kinase (AMPK) signaling cascade and the unc-51-like kinase 1 complex, oxidizing autophagy-related ATG4, disrupting the Bcl-2/Beclin-1 interaction, and inducing mitophagy, leading to changes in body homeostasis. Low GSH levels act as signals that activate autophagy in response to stress [82,83]. GSH is one of the main molecules in the thiol network that induces autophagy [83]. GSH deficiency triggers autophagy induction in germ cells as an adaptive stress response, independent of AMPK activation [82]. When GSH is depleted, H_2_O_2_ triggers autophagic cell death, enhances LC3 and p62/SQSTM1 degradation, and promotes autophagic vacuole production [84,85]. GSH redox homeostasis may play a central role in maintaining proteostasis by regulating autophagy [86].

Mitophagy is the selective degradation of mitochondria via autophagy. This is one of the most important mechanisms controlling mitochondrial quality and maintaining normal cellular homeostasis [87]. GSH regulates mitophagy independently of general autophagy. The antioxidant N-acetyl-cysteine (NAC) inhibits mitophagy but not autophagy. The effect of NAC on mitophagy involves its effect on GSH metabolism but not its clearance properties. Therefore, the regulation of intracellular GSH content can regulate mitophagy [88,89]. In addition, melatonin helps maintain mitochondrial function that is affected by glutamate-induced excitotoxicity [90]. It accomplishes this via ROS associated with mitophagy. Melatonin increases levels of SOD, GSH, and mitochondrial membrane potential while decreasing levels of GSSG and mtROS [90].

### 4.2. Induction of the Apoptotic Pathway

Apoptosis is a genetically controlled cascade of cell death processes characterized by membrane shrinkage, chromatin condensation, and apoptotic body formation, executed by a family of caspases [91]. Mitochondrial permeability transitions or pore formation by Bax and Bcl_2_ can lead to the release of apoptosis-inducing factors, the formation of apoptotic complexes, and the activation of caspases [92,93,94]. Depletion in GSH is a common feature of apoptosis, which can be triggered by various stimuli, such as the activation of death receptors, stress, environmental factors, and cytotoxic drugs.

The reduction in GSH due to ROS is transferred to the mitochondria, where it acts as a cofactor for glutathione peroxidase (GPx), converting H_2_O_2_ into H_2_O. As the levels of mGSH decrease, H_2_O_2_ continues to accumulate in the mitochondria and then diffuses out and starts causing cell damage. mtROS contribute to the oxidation of cardiolipin, which is controlled by antioxidants [79,95]. mGSH also indirectly regulates the redox state of cardiolipin and the release of apoptotic proteins via the mitochondrial permeability transition [79,96]. GSH depletion and the post-translational modification of proteins via glutathionylation are critical regulators of apoptosis [93]. Stressful conditions resulting from reduced mGSH levels can lead to the oxidation of the cardiolipin–cytochrome c complex. Consequently, cytochrome c diffuses out of the mitochondria through the pore created by Bax/Bak and triggers apoptosis via caspase-3 [97]. 

Increased ROS and an mGSH/GSSG imbalance can also stimulate intrinsic apoptotic pathways. Changes in the intracellular balance between GSH and GSSG play a significant role in determining the redox status and signaling of a cell [98]. GSSG has been shown to directly induce apoptosis by activating the stress-activated protein kinases/mitogen-activated protein kinase pathway [99]. Following mGSH depletion and caspase-3 activation, reduced mGSH levels induce ROS production and cytochrome c release [100]. ROS-induced ROS release can also promote apoptosis [101]. During apoptosis, depletion in GSH leads to a decrease in NADPH availability, which further contributes to a sustained cellular redox imbalance by impairing the reduction of GSSG by glutathione reductase [102]. Depletion of glutathione reductase results in GSH depletion and oxidative stress, ultimately making cells more susceptible to undergoing apoptosis [103].

Additionally, GSH depletion can occur via intrinsic or extrinsic apoptosis [104,105]. Reduced mGSH uptake affects mitochondrial function, leading to structural instability and the release of proapoptotic proteins [93]. Therefore, intracellular mGSH can be released to initiate or promote apoptosis, whereas the inhibition of GSH efflux during apoptosis can alleviate cell death [106]. The stimulation of GSH synthesis prevents the loss of mitochondrial membrane potential and inhibits apoptosis [107,108].

### 4.3. Induction of the Necroptosis Pathway

Although necrosis is a passive and unregulated form of cell death, some forms of necrosis, known as necroptosis, can be regulated by intracellular proteins [109,110]. Necroptosis exhibits unique features in mitochondria, lysates, and plasma membranes. These features include a translucent cytoplasm, swollen organelles, increased cell volume, and plasma membrane disruption [111,112]. Excessive ROS levels can induce apoptosis, whereas high ROS levels may lead to necroptosis. mGSH-depletion-induced ROS generation triggers apoptosis and necrosis. Cystine starvation or GSH degradation, which results in GSH depletion, can cause oxidative stress and lead to necroptosis and ferroptosis via direct lipid oxidation [113].

### 4.4. Induction of the Ferroptosis Pathway

Mitochondria play key roles in iron metabolism, heme synthesis, iron–sulfur protein assembly, and cellular iron regulation. Ferroptotic cells often exhibit mitochondrial swelling, a reduced number of cristae, mitochondrial membrane potential dissipation, and increased mitochondrial membrane permeability, indicating mitochondrial dysfunction [114]. Ferroptosis is characterized by excess intracellular iron and the accumulation of lethal lipid ROS [115]. Unlike mitochondrial fission, which promotes apoptosis, mitochondrial fusion occurs via the interferon-responsive cGAMP interactor 1–mitochondrial fusion 1/2 (MFN1/2) pathway [116]. It promotes mitochondrial oxidative damage and subsequent ferroptosis [117]. Mitochondrial energy metabolism is altered during ferroptosis, with increased OXPHOS and ATP production rates, resulting in decreased glycolysis rates [118]. Excessive oxidative stress can cause irreversible damage to mitochondria and reduce their integrity [119,120]. ROS production, mitochondrial membrane potential, mitochondrial fusion and fission, and mitophagy also play roles in ferroptosis [118,120].

Depletion in GSH in retinal pigment epithelium cells leads to ferroptosis via the combined mechanisms of ferroptosis and autophagy [85]. Ferroptosis stimulation attenuates mitochondrial bioenergetics and stimulates GSH depletion and GP_X_4 inactivation [121]. GP_X_4 reduces lipid hydroperoxides in biological membranes [122]. When GP_X_4 is deactivated, lipid ROS accumulates, leading to the initiation of ferroptosis [123]. Toxic lipids are involved in the dynamic pathways of lipid synthesis, degradation, storage, transformation, utilization, and peroxidation and directly mediate ferroptosis without involving pore-forming proteins [124,125]. Antioxidant systems and membrane repair pathways can synergistically antagonize organelle damage and ferroptosis caused by ROS [126,127,128,129,130,131]. When the mitochondrial membrane is rich in cholesterol, the activity of the GSH transport system is reduced [62]. Furthermore, mGSH mediates iron–sulfur cluster biogenesis rather than redox buffering [132]. HCBP6 reduces intracellular triglyceride levels via the SREBP1c/FASN pathway, and it interacts with and destabilizes SLC25A11, thereby affecting mGSH levels and regulating cardiac ferroptosis [133]. The inhibition of SLC25A10 and SLC25A11 increases mtROS and GSH consumption and aggravates myocardial ferroptosis [121]. In addition, the expression of crucial tumor suppressor gene TP53-induced glycolysis and the apoptosis regulator (TIGAR) was significantly increased in colorectal cancer tissues. Inhibiting TIGAR resulted in the downregulation of the GSH/GSSG ratio, increased lipid peroxidation, enhanced malondialdehyde (MDA) accumulation, and promoted ferroptosis [134].

## 5. GSH Deficiency and Disease

The levels of GSSG and GSH are indicators of cellular health. GSSG is harmful and needs to be converted back to GSH. An increase in GSH occurs in response to oxidative stress, and a decrease in GSH can worsen disease [78]. GSH deficiency plays an important role in various clinical conditions, mainly because of abnormalities in pathophysiological mechanisms [135]. Low GSH levels are a common pathway affecting all risk factors [136,137,138]. Disease progression may reduce the cellular uptake or synthesis of cysteine, leading to increased GSH efflux. Cysteine/glutathione sulfur loss occurs via accelerated oxidation to sulfates and taurine formation [139]. GSH acts as a primary defense mechanism by reducing and inactivating toxic oxidative intermediates [135]. GSH loss occurs when it is conjugated to drugs, toxins, and other substances, and is excreted from the cell as GSH or acetylcysteine thiolates (conjugates). For example, when the acetaminophen dose exceeds a patient’s ROS tolerance, it can easily lead to acute liver failure and GSH depletion [135]. There is a growing number of examples demonstrating the contribution of mGSH to various diseases. In many pathological conditions, mGSH depletion is not only a result of disease progression but also a cause of organ failure. This is often associated with cholesterol-mediated changes in membrane dynamics. Notably, mitochondrial cholesterol has been identified as a significant regulator of mitochondrial outer membrane permeabilization in response to apoptotic stimuli [140].

### 5.1. GSH Levels Are Reduced in Chronic Diseases

Patients with hypertension, ischemic heart disease, atherosclerotic and coronary artery diseases, diabetes, chronic lung disease, smoking, and obesity have reduced GSH levels and low GSH/GSSG ratios [136,137]. Reduced GSH levels are also observed in liver diseases, such as non-alcoholic fatty liver disease, alcoholic liver disease, ischemia/reperfusion injury, hepatitis C virus, hepatitis B virus, and hepatocellular carcinoma [141]. In addition, low GSH concentrations are associated with diseases such as asthma, chronic obstructive pulmonary disease, and atherosclerosis [142]. Furthermore, GSH depletion in the brain is associated with the onset and progression of neurodegenerative diseases such as Alzheimer’s and Parkinson’s diseases [143]. Genetic defects in GSH synthesis or homeostasis can affect GSH synthase, leading to hemolytic anemia, progressive neurological symptoms, metabolic acidosis, and, in severe cases, neonatal death [144]. 

### 5.2. GSH Levels Are Reduced in Microbial Infections

Reduced GSH levels have also been observed in diseases associated with inflammation caused by microbial infections. ROS and cysteine/glutathione depletion can lead to inflammatory responses, resulting in capillary leakage and organ failure [145]. Increasing age is associated with decreased GSH levels due to extensive oxidation of GSH and/or a reduction in cellular thiol pools [146,147,148]. Patients infected with *Plasmodium malaria* had lower levels of GSH-P_X_ [149] and GSH [150]. As human immunodeficiency virus infection progresses, the GSH levels in red blood cells, lymphocytes, and other peripheral blood mononuclear cells gradually decrease [151,152]. Patients infected with SARS-CoV-2 exhibit alterations in the glucose–insulin axis, including increased mitochondrial damage, oxidative/nitrosative stress, and significantly reduced levels of vitamin D, thiols, total antioxidant capacity (TAC), GSH, and selenium, resulting in hyperglycemia, hyperinsulinemia, and insulin resistance [153,154].

### 5.3. Diseases Related to GSH Enzyme Deficiency

In addition, GSTs are enzymes that catalyze the conjugation of GSH to various electrophilic substances. Changes or mutations in the genetic coding of GST isoenzymes, such as glutathione S-transferase Mu 1 (GSTM1) and glutathione S-transferase Theta 1 (GSTT1), result in decreased GSTM1 and GSTT1 enzymatic activity. The presence of the null GSTM1 or GSTT1 genotype may increase the risk of developing mitochondrial disease [155]. This decrease in activity is associated with higher levels of oxidative stress and can also impact susceptibility to diseases (Table 1). Deficiency in GSTM1 or GSTT1 is associated with an increased risk of various diseases (Table 1). Additionally, some individuals may lack the GSTM1 or GSTT1 gene, which can increase their vulnerability to certain diseases or affect their response to treatment. The deletion of the GSTM1 and GSTT1 genes may also vary among different races and ancestral backgrounds [156].

## 6. GSH Deficiency and Therapeutic Strategies

Maintaining the balance between GSH and GSSG in the mitochondria contributes to cellular health and prevents oxidative damage and programmed cell death, which are associated with aging and disease. GSH helps fight the oxidative stress caused by an imbalance between free radicals and antioxidants [170]. It can also reduce the severity and fatality associated with oxidative damage and inflammation [142]. mGSH functions directly or as a cofactor in reactions catalyzed by other mitochondrial enzymes to prevent or repair oxidative modifications that lead to mitochondrial dysfunction and cell death [37]. 

### 6.1. Increase GSH Levels

Supplementation with synthetic GSH or amino acids such as cysteine, glycine, and glutamic acid can also increase GSH levels. Supplementation with cysteine-rich whey protein increases GSH levels [171]. Glycine supplementation improves energy metabolism in damaged cells and increases ATP and GSH contents [172]. NAC is the precursor of cysteine and is deacetylated by N-deacetylase, which can increase intracellular cysteine levels, thereby increasing the endogenous synthesis of GSH [173]. The use of NAC or NAC with glycine can increase the intracellular GSH content, and more noticeable results were observed when glycine was added [174]. Supplementation with glutamine, a precursor of glutamate, increases intracellular GSH levels and protects cells from damage [173]. 

GSH neutralizes ROS overproduction during persistent tuberculosis infection. NAC supplementation may restore cellular GSH levels and prevent apoptosis caused by GSH depletion [175]. Appropriate doses of NAC can enhance pulmonary recovery and replenish the GSH levels in patients with respiratory diseases [176]. NAC supplementation can regulate GSH homeostasis, exert antioxidant effects, and improve athletic performance [177]. Glutathione-like liposomal GSH, when administered orally, intravenously, intranasally, or sublingually, increases erythrocyte and plasma GSH levels and reduces oxidative stress [173,178]. Meanwhile, NAC increases the activities of GSH and GSH-P_X_ and can reduce lipid peroxidation in the mitochondria [179]. 

### 6.2. Maintain the Ratio between GSH and GSSG

Increased levels of mtROS induce apoptosis and promote ferroptosis [114,126]. The accumulation of mitochondrial lipid ROS during ferroptosis may be partly due to mGSH depletion [121]. The mild uncoupling of modulated mitochondrial OXPHOS may serve as a potential therapeutic strategy for modulating intracellular ROS levels [180]. For example, fatty acids act as mild natural uncouplers [181] and can pass through the mitochondrial inner membrane in their protonated form and deprotonate on the matrix side. The anions of the fatty acids return to the cytoplasm to complete the cycle. The antioxidant enzyme microsomal glutathione S-transferase 1 is located in the mitochondria and ER and limits lipid peroxidation by binding to arachidonate 5-lipoxygenase [182].

Mitochondria-targeted ROS scavengers, such as mitochondrial superoxide scavengers and mitochondrial quinone, can inhibit ferroptosis [183,184]. In addition, mitochondrial antioxidant enzymes play crucial roles in ferroptosis inhibition. MnSOD is located in the mitochondrial matrix of eukaryotes and various prokaryotes and can block mtROS and induce ferroptosis in cancer cells [185]. For example, GP_X_4 is localized in the cytoplasm and mitochondrial intermembrane space [186] and helps mitigate mitochondrial oxidative damage during cell death, including ferroptosis [187]. Furthermore, mitochondrial dihydroorotate dehydrogenase mediates the oxidation of dihydroorotate to orotic acid, which is associated with the reduction of CoQ10 to ubiquinol, thereby limiting mitochondrial lipid peroxidation caused by GP_X_4 downregulation [129]. 

### 6.3. Related Adjunctive Therapies or Supplements Increase Antioxidant Effects

Some evidence suggests that dietary supplements are associated with increased GSH levels and antioxidant effects, as well as inflammation biomarker levels. Probiotics can increase GSH levels, thereby reducing inflammation and oxidative stress levels [188,189] (Table 2). Traditional Chinese medicines may also have some anti-inflammatory effects, and their potential mechanisms target antioxidant, anti-inflammatory, and apoptosis-related pathways. For example, ginsenosides affect oxidative-stress-related indicators, such as SOD, MDA, GSH, GSH-P_X_, and catalase, and reduce inflammatory factor levels [190]. A systematic review and meta-analysis showed that saffron supplementation can significantly increase TAC and GSH-P_X_ levels, suggesting that saffron may reduce oxidative stress markers [191]. The main mechanism of the neuroprotective effect of moringa extract and its phytochemical derivatives is to reduce oxidative stress by increasing antioxidant enzyme levels, reducing TAC levels, and inducing SOD and GSH overproduction [192]. In addition, a systematic review and meta-analysis showed that chromium supplementation significantly increases GSH levels [193]. The anti-inflammatory and antioxidant properties of zinc may also have broad therapeutic effects in cardiovascular diseases. Zinc supplementation significantly reduced nitric oxide, MDA, TAC, and GSH levels [194] (Table 3).

## 7. Conclusions

The mitochondria act as aerobic energy generators in eukaryotic cells. OXPHOS involves the transfer of electrons from the energy substrate to oxygen for ATP synthesis. However, the electron flow in the respiratory chain generates ROS, which can damage cells when excessive accumulation occurs. Mitochondria play a protective role in cells. Several approaches toward mitigating ROS-related damage have been developed. Mitochondrial oxidative stress and mGSH depletion are involved in many pathological conditions associated with mitochondrial abnormalities and dysfunction, as well as with disease and aging. Restoring mGSH levels and the GSH/GSSG ratio, as well as reducing ROS accumulation are critical for maintaining mitochondrial function and antioxidant defense. However, mGSH-related therapeutics require further research. Future studies may provide evidence that helps treat and prevent diseases caused by oxidative stress or the progression and oncogenic mitochondria.

## Figures and Tables

**Table 1:** Association between null mutations in glutathione S-transferase gene M1 and T1 and disease risk.

Gene	Disease	Difference (95% CI; *p*-Value)	Study
GSTM1	CKD	1.32 (1.12, 1.56; *p* = 0.0009)	Peng et al., 2023 [157]
	NAFLD	1.46 (1.20, 1.7; *p* = 0.0002)	Zhu et al., 2022 [158]
	Arsenic poisoning	0.731 (0.536, 0.999; *p* = 0.049)	Jin et al., 2021 [159]
	Lung adenocarcinoma	1.35 (1.22, 1.48)	Zhang et al., 2021 [160]
	Asthma	1.21 (1.07, 1.35; *p* < 0.001)	Su et al., 2020 [161]
	Low birth weight *	1.27 (1.12, 1.45)	Qu et al., 2020 [162]
	T2DM	1.37 (1.10, 1.70)	Nath et al., 2019 [163]
	Breast cancer	1.19 (1.03, 1.36)	Miao et al., 2019 [164]
	Leukemia	1.28 (1.16, 1.41; *p* < 0.0001)	Wang et al., 2019 [165]
	Male infertility	1.35 (1.02, 1.78)	Hu et al., 2019 [166]
	COPD	1.52 (1.31, 1.77; *p* < 0.00001)	Ding et al., 2019 [167]
	IBD	1.37 (1.13, 1.65; *p* = 0.001)	Zhou et al., 2019 [168]
GSTT1	CKD	1.52 (1.21, 1.90; *p* = 0.0003)	Peng et al., 2023 [157]
	NAFLD	1.34 (1.06, 1.68; *p* = 0.01)	Zhu et al., 2022 [158]
	Arsenic poisoning	1.009 (0.856, 1.189; *p* = 0.915)	Jin et al., 2021 [159]
	Lung adenocarcinoma	1.36 (1.17, 1.58)	Zhang et al., 2021 [160]
	Asthma	1.61 (1.30, 2.00; *p* < 0.001)	Su et al., 2020 [161]
	Low birth weight *	1.19 (0.97, 1.46)	Qu et al., 2020 [162]
	T2DM	1.29 (1.04, 1.61)	Nath et al., 2019 [163]
	Breast cancer	1.17 (1.05, 1.31)	Miao et al.,2019 [164]
	Osteosarcoma	1.247 (1.020, 1.524; *p* = 0.031)	Moghimi et al., 2019 [169]
	Leukemia	1.22 (1.07, 1.40; *p* = 0.003)	Wang et al., 2019 [165]
	Male infertility	1.40 (1.15, 1.70)	Hu et al., 2019 [166]
	COPD	1.28 (1.09, 1.50; *p* = 0.003)	Ding et al., 2019 [167]

* Maternal GSTM1 or GSTT1 null. COPD, chronic obstructive pulmonary disease; CKD, chronic kidney disease; GSTM1, glutathione S-transferase Mu 1; GSTT1, glutathione S-transferase Theta 1; IBD, inflammatory bowel disease; NAFLD, nonalcoholic fatty liver disease; T2DM, type 2 diabetes mellitus.

**Table 2:** Effects of probiotic intake on glutathione in different subjects.

**Study**	**Subjects**	Weighted Mean Difference * (95% CI)		***p*-Value**
Naseri et al., 2023 [188]	T2DM	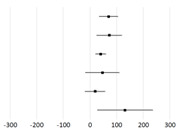	69.8 (33.65, 105.95)	<0.001
Dai et al., 2022 [195]	DKD		72.74 (24.19, 121.28)	0.003
Pourrajab et al., 2022 [196]	Adults		40.38 (20.72, 60.03)	<0.001
Amirani et al., 2020 [197]	PD		46.79 (−17.25, 110.83)	
Zamani et al., 2020 [198]	Adults		19.32 (−18.7, 57.33)	
Roshan et al., 2019 [199]	Adults		132.36 (27.76, 236.95)	0.01
**Study**	**Subjects**	Standardized Mean Difference * (95% CI)		***p*-Value**
Tabrizi et al., 2022 [200]	POS	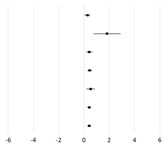	0.26 (0.01, 0.52)	0.04
Xu et al., 2021 [201]	ALI		1.83 (0.76, 2.91)	0.01
Nguyen et al., 2021 [202]	HD		0.4 (0.14, 0.66)	0.003
Zheng et al., 2021 [203]	CKD		0.44 (0.25, 0.65)	0
Bakhtiary et al., 2021 [204]	CKD		0.52 (0.19, 0.86)	0.19
Ardeshirlarijani et al., 2019 [205]	T2DM		0.41(0.26, 0.56)	0.182
Zheng et al., 2019 [206]	DM		0.41 (0.26, 0.55)	0
**Study**	**Subjects**	**Mean Difference (95% CI)**		***p*-Value**
Kou et al., 2023 [207]	Elderly	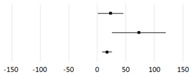	17.08 (8.65, 25.5)	<0.01
Tan et al., 2022 [208]	CKD		72.86 (25.44, 120.29)	
Zhang et al., 2019 [209]	Gestational DM		23.13 (0.65, 45.62)	0.04

* The raw mean difference can be scaled with the inverse variance weight to define the weighted mean difference. The mean standardized difference quantifies the difference between group means using one or more variables. ALI, acute liver injury; CKD, chronic kidney disease; DKD, diabetic kidney disease; DM, diabetes mellitus; HD, hemodialysis; PD, psychiatric disorder; POS, polycystic ovary syndrome; T2DM, type 2 diabetes mellitus.

**Table 3:** The clinical effectiveness of zinc supplementation on glutathione.

Study	Subjects	Difference * (95% CI)	*p*-Value
Faghfouri et al., 2021 [210]	Adults	SMD 1.28 (0.42, 2.14)	0.003
Mohammadi et al., 2021 [211]	Adults	WMD: 34.84 (−5.12, 74.80)	0.087
Jimenez-Fernandez et al., 2021 [212]	BD	SMD = 2.49 (0.58, 4.39)	0.01
Mousavi et al., 2020 [213]	Adults	MD: 49.99 (2.25–97.73)	

* The raw mean difference can be scaled with the inverse variance weight to define the weighted mean difference. The mean standardized difference quantifies the difference between group means using one or more variables. BD, bipolar disorder; MD, mean difference; SMD, standardized mean difference; WMD, weighted mean difference.

## Data Availability

No new data were created or analyzed in this study. Data sharing does not apply to this article.
